# Secular trends in pediatric antiretroviral treatment programs in rural and urban Zambia: a retrospective cohort study

**DOI:** 10.1186/1471-2431-10-54

**Published:** 2010-07-30

**Authors:** Catherine G Sutcliffe, Carolyn Bolton-Moore, Janneke H van Dijk, Matt Cotham, Bushimbwa Tambatamba, William J Moss

**Affiliations:** 1 Bloomberg School of Public Health, Johns Hopkins University, 615 North Wolfe Street, Baltimore, MD, USA, 21205; 2 Centre for Infectious Disease Research in Zambia, 5977 Benakale Road, Northmead, Lusaka, Zambia; 3 Macha Research Trust, Macha Hospital, PO Box 630 166, Choma, Zambia; 4 Mukinge Hospital, PO Box 120092, Kasempa, Zambia; 5 Ministry of Health, Lusaka, Zambia

## Abstract

**Background:**

Since 2003 pediatric antiretroviral treatment (ART) programs have scaled-up in sub-Saharan Africa and should be evaluated to assess progress and identify areas for improvement. We evaluated secular trends in the characteristics and treatment outcomes of children in three pediatric ART clinics in urban and rural areas in Zambia.

**Methods:**

Routinely collected data were analyzed from three ART programs in rural (Macha and Mukinge) and urban (Lusaka) Zambia between program implementation and July 2008. Data were obtained from electronic medical record systems and medical record abstraction, and were categorized by year of program implementation. Characteristics of all HIV-infected and exposed children enrolled in the programs and all children initiating treatment were compared by year of implementation.

**Results:**

Age decreased and immunologic characteristics improved in all groups over time in both urban and rural clinics, with greater improvement observed in the rural clinics. Among children both eligible and ineligible for ART at clinic enrollment, the majority started treatment within a year. A high proportion of children, particularly those ineligible for ART at clinic enrollment, were lost to follow-up prior to initiating ART. Among children initiating ART, clinical and immunologic outcomes after six months of treatment improved in both urban and rural clinics. In the urban clinics, mortality after six months of treatment declined with program duration, and in the rural clinics, the proportion of children defaulting by six months increased with program duration.

**Conclusions:**

Treatment programs are showing signs of progress in the care of HIV-infected children, particularly in the rural clinics where scale-up increased rapidly over the first three years of program implementation. However, continued efforts to optimize care are needed as many children continue to enroll in ART programs at a late stage of disease and thus are not receiving the full benefits of treatment.

## Background

At the end of 2008, approximately 3 million people were receiving antiretroviral therapy (ART) in sub-Saharan Africa, including over 200,000 children, representing a substantial increase in coverage for those in need since the World Health Organization (WHO) announced its '3 by 5' initiative in 2003 [1,2]. Recent studies have demonstrated that children receiving ART in sub-Saharan Africa can achieve comparable outcomes to children in high-income countries [3]. However, higher mortality rates have been observed due to treatment initiation at more advanced stages of disease [4]. As treatment programs scale-up and more HIV-infected women receive prevention of mother-to-child transmission (PMTCT) services, awareness of the availability of testing and treatment services for infants and children should increase. This increased awareness should be accompanied by improvements in the profile of children enrolling in ART programs, as infants and children are brought for testing and treatment earlier, prior to the development of signs and symptoms of advanced disease. In studies among adults initiating ART in sub-Saharan Africa, baseline CD4^+ ^T-cell counts increased with increasing duration of program implementation [5-7], and mortality rates post-ART initiation decreased [7]. However, several studies found increases in loss-to-follow-up as programs expanded and are burdened with tracking growing patient populations [7,8].

Evaluation of pediatric ART programs over time can be an effective method to assess the progress of these programs, not only by characterizing the age, stage of disease and outcomes of HIV-infected children accessing treatment services, but also by ascertaining the number of HIV-exposed children enrolled, which provides a measure of how well pediatric and maternal health programs within healthcare facilities are collaborating to identify HIV-exposed infants.

Monitoring and evaluating pediatric ART programs in different settings, including rural and urban areas, will also be important, as rural clinics may face different challenges at the level of the provider and caregiver, including shortages of healthcare workers, drugs or laboratory equipment and greater travel distances to the clinic [9,10]. These factors could affect the characteristics of the patient population as well as treatment outcomes over time. We evaluated secular trends in the characteristics and treatment outcomes of children in three pediatric ART clinics in urban and rural areas of Zambia between 2004 and 2008.

## Methods

### Study Population

The study was conducted in two rural and one urban pediatric HIV clinic in Zambia. The urban clinic, Matero Reference Clinic, is located in a low-income community in Lusaka and is one of 18 Ministry of Health facilities supported by the Centre for Infectious Disease Research in Zambia (CIDRZ) that has provided treatment to HIV-infected children since May 2004. Matero Reference Clinic has a similar pediatric patient population with comparable treatment outcomes to the other CIDRZ clinics in Lusaka [11].

The two rural clinics were Macha Mission Hospital and Mukinge Hospital. Macha Mission Hospital, located in Southern Province, is a district-level hospital administered by the Brethren in Christ Church serving a population of over 150,000 people [12,13]. Mukinge Hospital, located in Mukinge in North Western Province, is a district-level hospital administered by the Evangelical Church in Zambia serving a population of over 100,000 people. The HIV clinics in Macha and Mukinge function within the healthcare system of the Ministry of Health and began administering ART in March 2005. As faith-based health facilities, Macha and Mukinge may have different support and healthcare personnel than rural government clinics. Both rural areas have lower population densities than Lusaka (63.5 per km^2 ^in Lusaka, 14.2 per km^2 ^in Southern Province and 4.6 per km^2 ^in North Western Province), and are populated primarily by subsistence farmers [14].

The study was approved by the University of Zambia Research Ethics Committee, the Ministry of Health of Zambia, and the Institutional Review Board at the Johns Hopkins Bloomberg School of Public Health.

### Clinic Procedures

Clinic procedures were similar at all three clinics. Children with a positive serological test for HIV were eligible for enrollment. At the initial evaluation visit, children underwent a medical history, physical examination, anthropometric measurements, collection of socio-demographic information, and determination of WHO disease stage. Standard of care included measurement of CD4^+ ^T-cell counts and percentages (not available in Macha/Mukinge in the first year of the program), hemoglobin levels, and renal and liver function tests. Capacity to routinely measure HIV viral loads was not available. Staffing in the three clinics was similar, with 1-2 physicians, 1-2 clinical officers and several nurses and counselors in the clinic at any time.

Treatment eligibility was determined based on WHO [15,16] treatment guidelines in effect at the time. For HIV-seropositive infants younger than 18 months, DNA-based diagnostic testing methods were available in Lusaka in 2007. However, they were not widely used by public-sector health clinics until 2008 and only became available in Macha and Mukinge in February 2008. Consequently, infection in these children could not be laboratory-confirmed during the study period. HIV-seropositive infants diagnosed with severe disease or immunodeficiency were presumptively treated, as recommended by WHO treatment guidelines [16].

Children determined to be eligible were treated with a first-line regimen consisting of two nucleoside reverse transcriptase inhibitors (stavudine or zidovudine or abacavir plus lamivudine) and one non-nucleoside reverse transcriptase inhibitor (efavirenz or nevirapine), and asked to return for clinical evaluation every three months. Laboratory measurements were performed every six months or when clinically indicated. Children not receiving ART returned for clinical and laboratory evaluation every three months. Attempts were made to trace children who missed scheduled appointments to ascertain whether they had died, moved or dropped out of the program (defaulted).

### Statistical Methods

**Figure 1:**
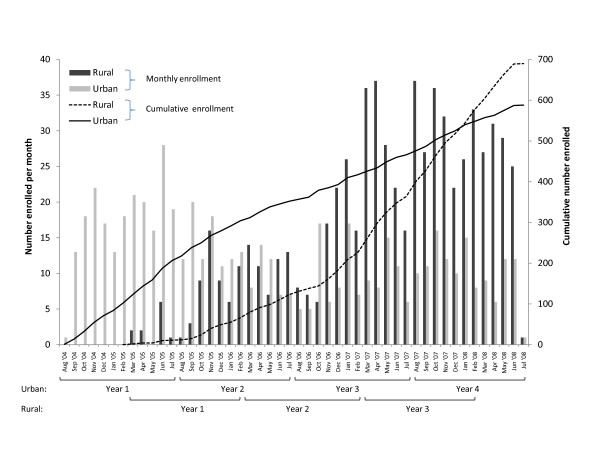
**Enrollment over time in three pediatric HIV programs in urban and rural Zambia**. Monthly enrollment of HIV-exposed and infected children in two rural and one urban HIV clinic in Zambia between August 2004 and July 2008.

Data were collected between program implementation and July 31, 2008 from the CAREWare electronic medical records system at Macha and Mukinge and from medical record abstraction at Matero Reference Clinic (95% of pediatric files were located). The duration of program implementation was divided into four years for Matero (Year 1: Aug 2004-July 2005; Year 2: Aug 2005-July 2006; Year 3: Aug 2006-Aug 2007; Year 4: Aug 2007-July 2008) and three years for Macha and Mukinge (Year 1: Mar 2005-Feb 2006; Year 2: Mar 2006-Feb 2007; Year 3: Mar 2007-Feb 2008) (Figure 1). For analyses at the initial evaluation visit, all HIV-seropositive treatment-naïve children younger than 15 years and enrolled in the HIV programs during the specified years of program implementation were included. When comparing HIV-related characteristics at the initial evaluation visit, the analysis was restricted to children with confirmed (≥18 months old) or presumed (<18 months old) infection. Children younger than 18 months at enrollment who were presumptively diagnosed and treated or who were older than 18 months of age with a positive serological test by July 2008 were presumed to be HIV-infected at enrollment. Severe immunodeficiency was defined by age-specific CD4^+ ^T-cell percentages or CD4^+ ^T-cell counts according to the 2006 WHO guidelines [16]. Eligibility for ART at clinic enrollment was defined retrospectively based on the treatment guidelines in effect at the time the child enrolled in the clinic (the 2003 guidelines were assumed to have been in effect until June 1, 2006). As the 2003 guidelines relied more heavily on clinical judgment for certain groups, eligibility was defined as initiating ART within 90 days of enrollment for children with ambiguous or missing WHO stage and CD4^+ ^T-cell percentage. Laboratory measures within 90 days were used if none were available from the initial evaluation visit.

Outcomes for HIV-infected children were determined one year after enrollment and were evaluated by eligibility for ART at the initial evaluation visit. Outcomes included initiating ART and remaining in care, and transferring to another clinic, dying or defaulting without initiating ART. Children who missed at least two clinic visits scheduled three months apart by the end of follow-up (i.e., had not been seen in the clinic since January 1, 2008) were assumed to have defaulted and were censored at their last visit. As outcomes were defined after one year in the clinic, the last year of program implementation was excluded from this analysis.

For time trends after ART initiation, the analysis was restricted to all children younger than 15 years with confirmed or presumed HIV-infection who initiated ART during the specified years of program implementation. Characteristics at ART initiation were evaluated, including laboratory measures within 90 days prior to ART initiation. The child's status was determined after six months on ART. Clinical and immunologic outcomes were assessed at six months post-ART initiation. Immunologic outcomes were assessed by determining the median CD4^+ ^T-cell percentage (both absolute and change from ART initiation) at the six month visit. Clinical outcomes were assessed by determining the proportion of children who were underweight at the six month visit. Weight-for-age z-scores (WAZ) were used to determine nutritional status and were calculated using the WHO growth standards as a reference and statistical software provided by WHO [17]. Children with WAZ scores below -2 were considered to be underweight. For this analysis, the CD4^+ ^T-cell percentage and WAZ closest to six months after initiation of ART (+/- 2 months) was used.

Mantel-Haenszel χ^2 ^and Cuzick's non-parametric test were used to test for trends in categorical and continuous variables, respectively. Analyses were conducted using SAS for Windows, version 9.1 (SAS Institute Inc, Cary, North Carolina) and STATA, version 9 (StataCorp LP, College Station, Texas).

## Results

### Changes in the characteristics of children at the initial evaluation over time

#### All children enrolled in the clinics

During the study period, 588 and 690 children were enrolled in the urban and rural (606 in Macha and 84 in Mukinge) clinics, respectively (Figure 1). In the urban clinic, the number of children enrolled decreased over time. There was a non-significant trend towards decreasing age at enrollment over time (Table 1); however, the median age remained high in the fourth year of implementation. No differences in enrollment by gender or township of residence were observed over time.

**Table 1:** Characteristics at initial evaluation at an HIV clinic in Lusaka, Zambia, by year of enrollment

	First Year N (%)	Second Year N (%)	Third Year N (%)	Fourth Year N (%)	p-trend^a^
All children	n = 206	n = 146	n = 114	n = 122	

Age (yrs): Median (IQR)	6.9 (3.4, 10.0)	5.9 (2.5, 9.4)	5.6 (2.5, 9.1)	5.5 (1.8, 10.3)	0.12
< 1	12 (5.8)	4 (2.7)	5 (4.4)	13 (10.7)	0.14
1-1.9	25 (12.1)	23 (15.8)	17 (14.9)	20 (16.4)	
2-4.9	45 (21.8)	34 (23.3)	30 (26.3)	21 (17.2)	
5-9.9	70 (34.0)	57 (39.0)	38 (33.3)	37 (30.3)	
≥ 10	54 (26.2)	28 (19.2)	24 (21.1)	31 (25.4)	
Male sex	111 (54.2)	74 (50.7)	56 (49.6)	64 (52.9)	0.71
Resides in Matero Township	66 (32.2)	51 (35.2)	38 (33.3)	40 (33.1)	0.90

HIV-infected children	n = 199	n = 140	n = 109	n = 114	

Age (yrs): Median (IQR)	7.1 (3.6, 10.2)	6.4 (2.9, 9.6)	6.0 (3.1, 9.5)	6.1 (2.2, 10.5)	0.24
Male sex	107 (54.0)	72 (51.4)	55 (50.9)	61 (53.5)	0.84
Self-reported history of tuberculosis	81 (42.4)	37 (26.6)	32 (30.8)	22 (20.8)	0.0003
Missing	8 (4.0)	1 (0.7)	5 (4.6)	8 (7.0)	
WAZ: Median (IQR)	-2.0 (-3.1, -1.0)	-2.5 (-3.8, -1.8)	-1.9 (-3.1, -1.0)	-2.7 (-4.1, -1.5)	
Underweight	70 (49.7)	75 (70.1)	38 (48.1)	44 (62.0)	0.34
Missing	58 (29.1)	33 (23.6)	30 (27.5)	43 (37.7)	
WHO stage					
1	34 (17.3)	7 (5.0)	10 (9.2)	14 (12.3)	<0.0001
2	82 (41.6)	23 (16.6)	22 (20.2)	19 (16.7)	
3 or 4	81 (41.1)	109 (78.4)	77 (70.6)	81 (71.1)	
Missing	2 (1.0)	1 (0.7)	0 (0.0)	0 (0.0)	
CD4%: Median (IQR)	11.9 (5.8, 17.5)	13.3 (6.9, 19.4)	13.2 (8.3, 18.8)	15.1 (9.1, 21.9)	0.001
Missing	24 (12.1)	13 (9.3)	4 (3.7)	4 (3.5)	
Severe immunodeficiency^b^	120 (66.3)	75 (59.1)	66 (62.9)	64 (58.2)	0.22
CD4 count (cells/mm^3^): Median (IQR)	356 (132, 603)	328 (135, 531)	439 (214, 611)	429 (209, 766)	0.004
Missing	19 (9.5)	13 (9.3)	4 (3.7)	4 (3.7)	
Eligible for ART^c^	146 (73.4)	122 (87.1)	92 (84.4)	95 (83.3)	0.02

IQR: interquartile range; WAZ: Weight-for-age z-score (only for children younger than 10 years); WHO: World Health Organization; ART: antiretroviral therapy.

^a ^p-trend calculated from Mantel-Haenszel χ^2 ^for categorical variables and Cuzick's non-parametric test for trend for continuous variables.

^b ^severe immunodeficiency defined by age according to the 2006 World Health Organization guidelines.

^c ^determined retrospectively based on the WHO guidelines in effect at the time of the initial evaluation

In the rural clinics, enrollment increased over time (Table 2), largely due to an increase in the number of HIV-exposed children in Macha, which lead to a significant decrease in the age at enrollment. No differences in enrollment by gender were observed over time. The median distance travelled to the clinic also significantly increased over time.

**Table 2:** Characteristics of HIV-exposed and infected children at initial evaluation in two rural clinics in Zambia by year of enrollment

	First Year N (%)	Second Year N (%)	Third Year N (%)	p-trend^a^
All children	N = 66	n = 159	n = 352	

Age (yrs): Median (IQR)	3.1 (1.0, 8.1)	1.7 (0.6, 5.2)	0.4 (0.0, 1.9)	<0.0001
<1	17 (25.8)	57 (35.9)	224 (63.6)	<0.0001
1-1.9	9 (13.6)	31 (19.5)	46 (13.1)	
2-4.9	17 (25.8)	26 (16.4)	38 (10.8)	
5-9.9	14 (21.2)	29 (18.2)	31 (8.8)	
≥ 10	9 (13.6)	16 (10.1)	13 (3.7)	
Male sex	34 (51.5)	68 (42.8)	182 (51.7)	0.38
Distance travelled (km):				0.008
Median (IQR)	22 (9, 40)	30 (13, 48)	35 (20, 50)	
Missing	11 (16.7)	41 (25.8)	80 (22.7)	

HIV-infected children	N = 48	N = 99	n = 124	

Age (yrs): Median (IQR)	4.9 (2.6, 9.3)	3.9 (1.9, 7.5)	2.8 (1.7, 6.5)	0.003
Male sex	24 (50.0)	41 (41.4)	66 (53.2)	0.39
Self-reported history of tuberculosis	13 (27.1)	11 (11.1)	15 (12.1)	0.04
WAZ: Median (IQR)	-3.0 (-3.8, -2.3)	-2.3 (-4.1, -1.5)	-3.0 (-3.9, -1.5)	
Underweight	27 (81.8)	43 (57.3)	69 (70.4)	0.71
Missing	15 (31.3)	24 (24.2)	26 (21.0)	
WHO stage				
1	4 (10.8)	9 (11.5)	16 (18.8)	0.04
2	10 (27.0)	32 (41.0)	34 (40.0)	
3 or 4	23 (62.2)	37 (47.4)	35 (41.2)	
Missing	11 (22.9)	21 (21.2)	39 (31.5)	
CD4%: Median (IQR)	19.0 (9.0, 25.0)	13.0 (9.5, 19.4)	20.2 (13.7, 27.6)	0.01
Missing	41 (85.4)	50 (50.5)	45 (36.3)	
Severe immunodeficiency^b^	3 (25.0)	36 (60.0)	36 (41.9)	0.59
CD4 count (cells/mm^3^):				
Median (IQR)	453 (257, 663)	513 (264, 786)	693 (338, 998)	0.01
Missing	36 (75%)	39 (39.4)	38 (30.6)	
Eligible for ART^c^	22 (55.0)	58 (61.7)	61 (54.5)	0.69
Missing	8 (16.7)	5 (5.1)	12 (9.7)	

IQR: interquartile range; WAZ: Weight-for-age z-score (only for children younger than 10 years); WHO: World Health Organization; ART: antiretroviral therapy.

^a ^p-trend calculated from Mantel-Haenszel χ^2 ^for categorical variables and Cuzick's non-parametric test for trend for continuous variables.

^b ^severe immunodeficiency defined by age according to the 2006 WHO guidelines.

^c ^determined retrospectively based on the WHO guidelines in effect at the time of the initial evaluation

#### HIV-infected children

Similar non-significant decreases in age at enrollment were observed among HIV-infected children in the urban clinic (Table 1). In addition, the proportion of children with a history of tuberculosis significantly decreased over time. In contrast, the proportion of children with severe immunodeficiency remained stable and the proportion of children presenting with WHO stage 3 or 4 disease increased significantly over time, resulting in a significant increase in the proportion of children who were eligible for ART at the initial evaluation visit.

Among HIV-infected children in the rural clinics, the median age was lower than that of urban children and decreased significantly over time (Table 2). The children's clinical status also significantly improved over time, with the proportion reporting a history of tuberculosis and WHO stage 3 or 4 decreasing over time.

The availability of laboratory tests increased over time in the rural clinics, with few children undergoing tests to measure immune status in the first year of the program. In the second and third years of program implementation, when at least half of the children had measures available, the proportion with severe immunodeficiency decreased, resulting in a decrease in the proportion of children eligible for ART at initial evaluation.

### Changes in outcomes among HIV-infected children eligible and ineligible for ART at clinic enrollment

In both urban and rural clinics, no significant changes over time in outcomes were observed for children who were eligible and ineligible for ART at enrollment (Table 3). The majority of children who were eligible initiated ART within a year but reasons for remaining in care without initiating ART were not available. Among eligible children who did not remain in care, a small proportion died or transferred to other clinics but the majority (approximately 10% of all eligible children) were lost to follow-up.

**Table 3:** One year retention and outcomes by ART eligibility status at initial evaluation among HIV-infected children in urban and rural Zambia

	First year N (%)	Second year N (%)	Third year N (%)	p-trend^a^
Urban				

Eligible for ART^b^	N = 146	N = 122	N = 92	0.39
On ART	131 (89.7)	99 (81.2)	78 (84.8)	
Alive, not on ART	3 (2.1)	3 (2.5)	3 (3.3)	
Died	1 (0.7)	3 (2.5)	1 (1.1)	
Defaulted	11 (7.5)	17 (13.9)	9 (9.8)	
Transferred	0 (0.0)	0 (0.0)	1 (1.1)	
Ineligible for ART^b^	N = 53	N = 18	N = 17	0.87
On ART	20 (37.7)	9 (50.0)	4 (23.5)	
Alive, not on ART	17 (32.1)	5 (27.8)	6 (32.3)	
Died	0 (0.0)	1 (5.6)	1 (5.9)	
Defaulted	14 (26.4)	3 (16.7)	5 (29.4)	
Transferred	2 (3.8)	0 (0.0)	1 (5.9)	

Rural				

Eligible for ART^b^	N = 22	N = 58		0.29
On ART	18 (81.8)	33 (56.9)		
Alive, not on ART	2 (9.1)	17 (29.3)		
Died	0 (0.0)	0 (0.0)		
Defaulted	2 (9.1)	8 (13.8)		
Transferred	0 (0.0)	0 (0.0)		
Ineligible for ART^b^	N = 18	N = 36		0.63
On ART	9 (50.0)	7 (19.4)		
Alive, not on ART	6 (33.3)	16 (44.4)		
Died	0 (0.0)	2 (5.6)		
Defaulted	3 (16.7)	11 (30.6)		
Transferred	0 (0.0)	0 (0.0)		

^a ^p-trend calculated from Mantel-Haenszel χ^2^

^b ^determined retrospectively based on the WHO guidelines in effect at the time of the initial evaluation

Among children ineligible for ART at enrollment, up to 50% initiated ART within one year and approximately 30% remained alive and in care. Of those children who did not remain in care, the majority (up to 30% of all ineligible children) were lost to follow-up.

### Changes in outcomes among HIV-infected children receiving ART

**Figure 2:**
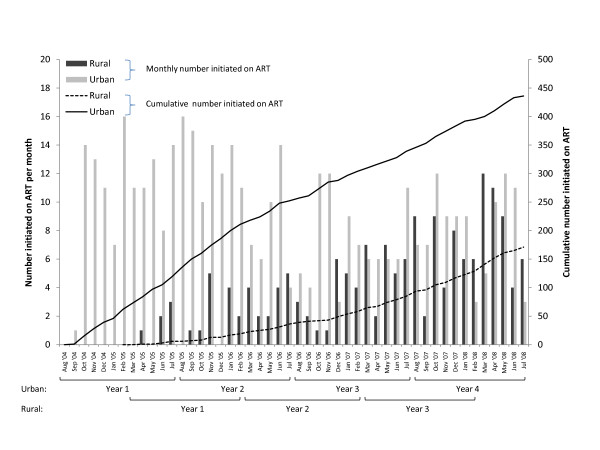
**Number of children initiating ART over time in three pediatric HIV programs in urban and rural Zambia**. Monthly and cumulative number of HIV-infected children initiating ART in two rural and one urban HIV clinic in Zambia between August 2004 and July 2008.

During the study period, 436 and 171 children started ART in the urban and rural clinics, respectively (Figure 2). At ART initiation, changes over time in age and CD4^+ ^T-cell percentage in both urban and rural clinics were similar to those observed at enrollment, with decreases in age and increases in CD4^+ ^T-cell percentage at ART initiation (Table 4). Among children on ART in the urban clinic, there were no significant differences over time in the proportion of children who transferred to other ART programs or who defaulted after six months on ART (Table 3). There was a significant decrease in the proportion of deaths within six months of initiating ART, resulting in an increase in the proportion of children remaining in care over time. Immunologic outcomes improved, although trends were not statistically significant. Clinical outcomes, in terms of the proportion of underweight children, initially worsened and then improved in the fourth year of the program.

**Table 4:** Retention and outcomes for HIV-infected children in the first 6 months of antiretroviral therapy in Zambia, by year of enrollment

	First Year N (%)	Second Year N (%)	Third Year N (%)	Fourth Year N (%)	p-trend^a^
Urban	n = 119	n = 133	n = 87	N = 97	

At ART initiation					
Age (yrs): Med (IQR)	7.4 (3.6, 10.6)	6.8 (3.0, 9.7)	7.0 (3.7, 9.9)	6.0 (2.4, 9.1)	0.21
CD4%: Med (IQR)	8.8 (4.8, 12.7)	11.6 (6.8, 17.5)	11.4 (7.1, 16.0)	12.8 (9.0, 18.6)	<0.0001
Missing	11 (9.2)	17 (12.8)	4 (4.6)	3 (3.1)	
At 6 months					
Still receiving care	90 (75.6)	104 (78.2)	67 (77.0)	86 (88.7)	0.03
Death	8 (6.7)	12 (9.0)	3 (3.5)	0 (0.0)	0.009
Transferred	7 (5.9)	7 (5.3)	7 (5.1)	4 (4.1)	0.81
Defaulted	14 (11.8)	10 (7.5)	10 (11.5)	7 (7.2)	0.44
Immunologic outcome	N = 49 (54.4)^b^	N = 67 (64.4)	N = 47 (70.2)	N = 33 (38.4)	
CD4%: Med (IQR)	22.7 (15.9, 27.8)	24.5 (19.7, 33.1)	22.7 (16.0, 28.5)	26.7 (22.4, 31.8)	0.15
Change in CD4%: Med (IQR)^c^	12.4 (8.1, 15.9)	13.3 (9.1, 17.7)	10.3 (4.2, 14.3)	12.8 (6.7, 16.6)	0.24
Clinical outcome	N = 48 (53.3)^b^	N = 74 (71.2)	N = 49 (73.1)	N = 35 (40.7)	
WAZ < -2	18 (37.5)	32 (43.2)	21 (42.9)	11 (31.4)	0.65

Rural	n = 19	n = 39	n = 71		

At ART initiation					
Age (yrs): Med (IQR)	8.1 (3.4, 10.1)	6.5 (1.9, 10.7)	3.1 (1.7, 7.3)		0.03
CD4%: Med (IQR)	9.0 (9.0, 9.0)	11.8 (8.0, 16.0)	13.7 (9.5, 18.4)		0.23
Missing	18 (94.7)	13 (33.3)	27 (38.0)		
At 6 months					
Still receiving care	16 (84.2)	31 (79.5)	51 (71.8)		0.21
Death	2 (10.5)	5 (12.8)	9 (12.7)		0.84
Transferred	1 (5.3)	2 (5.1)	3 (4.2)		0.81
Defaulted	0 (0.0)	1 (2.6)	8 (11.3)		0.04
Immunologic outcome	N = 4 (25.0)^b^	N = 13 (41.9)	N = 20 (39.2)		
CD4%: Med (IQR)	12.1 (10.6, 28.8)	19.4 (14.4, 33.1)	25.9 (18.2, 32.9)		0.22
Change in CD4%: Med (IQR)^c^	3.4 (3.4, 3.4)	7.7 (-2.4, 20.0)	12.6 (4.9, 21.0)		0.29
Clinical outcome	N = 12 (75.0)^b^	N = 18 (58.1)	N = 32 (62.8)		
WAZ < -2	8 (66.7)	10 (55.6)	16 (50.0)		0.33

ART: antiretroviral therapy; WAZ: Weight-for-age z-score (only for children younger than 10 years); IQR: interquartile range

^a ^p-trend calculated from Mantel-Haenszel χ^2 ^for categorical variables and Cuzick's non-parametric test for trend for continuous variable.

^b ^Numbers in parentheses represent the proportion of those receiving care at 6 months for whom a measure is available

^c ^Samples sizes are smaller than indicated due to missing data at ART initiation

Among children in the rural clinics, there were no significant differences over time in the proportion of children who died or transferred to another ART program after six months of ART (Table 4). However, the proportion of children who defaulted increased significantly over time, resulting in a decrease in the proportion of children remaining in care. Both immunologic and clinical outcomes after six months of ART improved over time, although these trends were not statistically significant.

## Discussion

In this study of trends in the characteristics of children enrolling in ART programs and initiating ART in urban and rural Zambia, signs of progress were evident in the first years of program implementation but differed between rural and urban clinics, reflecting differences not only in the characteristics of the population attending the clinics but also in program implementation, infrastructure and integration. In evaluating characteristics of HIV-infected children enrolling in ART programs, we were able to examine the success of these programs in improving the availability of ART and promoting testing of children at younger ages and earlier stages of disease progression. The older age and later stage of disease of HIV-infected children observed in this study, in particular in the urban clinic, are consistent with other studies of HIV-infected children in sub-Saharan Africa [3,18], and resulted in the majority of children being eligible for ART at the time of enrollment. Concerns have been raised that treatment is initiated late in disease progression, reducing its effectiveness [19]. Efforts to identify HIV-infected children earlier should result in treatment before signs and symptoms of severe disease.

In comparison to the urban clinics, and contrary to studies among adults initiating ART in urban and rural areas [20,21], children in the rural clinics appeared to enroll in care and initiate ART at younger ages and earlier stages of disease, and to have greater improvements in these measures over time. It is encouraging that despite the many obstacles to care in rural areas, these rural clinics have been able to address these issues and make improvements, which may translate into better treatment outcomes [3,11].

We not only examined trends among HIV-infected children but also among all children, both HIV-exposed and HIV-infected, enrolled in the clinics. As treatment and PMTCT programs scale-up and awareness increases about HIV/AIDS and testing and treatment services, ART clinics will see an increase in the number of enrolled HIV-exposed children. This pattern was observed in the rural clinics, where the number of children enrolled increased more than five times in the first three years, largely due to an increase in the number of HIV-exposed children. Increased awareness in the community is supported by the observed increase in the distances travelled to the clinic over time.

These patterns were not observed in the urban clinic, where enrollment decreased over time and few infants were enrolled. These differences in age distribution can be attributed to differing procedures, with the rural clinics having more active enrollment procedures than the urban clinic. In the rural clinics, HIV-exposed children were tested and referred to the ART clinic from maternal and child health and pediatric programs and were followed until their HIV status could be confirmed. In the urban clinic, there was less collaboration between programs and higher attrition among referrals [22], such that many infants were either not tested or contact was lost before they obtained their test results and could be referred to the ART clinic. Under this system, the onus was on the caregiver to seek testing, care and treatment for their child.

These findings have implications for treatment programs in light of recent revisions to the treatment guidelines, recommending treatment of HIV-infected children younger than 2 years [23] following the results of the Children with HIV Early Antiretroviral Therapy (CHER) trial in South Africa [24]. Implementation of these recommendations requires that HIV-exposed children be identified and infection confirmed in order to initiate treatment, a process that appears to be working well in the rural clinics where a large group of HIV-exposed children are enrolled and can benefit from the new treatment guidelines. Similar progress has not been achieved in the urban clinic where continued work is needed to promote HIV testing and collaboration, and integration of pediatric, nutrition, and maternal health programs within healthcare facilities. As more infants are enrolled into these programs, further research will be needed on how well these children respond to treatment, as much of the data on clinical, immunologic, and virologic outcomes from sub-Saharan Africa come from studies of older children.

Outcomes among eligible and ineligible children did not vary over time but demonstrated that these programs were successfully starting children on treatment, as the majority of eligible and ineligible children in both urban and rural clinics initiated ART within a year of clinic enrollment. Fewer eligible children appeared to initiate ART in the rural clinics, possibly due to the high levels of malnutrition in these areas. Severe malnutrition is an indicator for severe disease (WHO stage 3) but by itself would not indicate eligibility for ART. A major concern was the high proportion of children lost to follow-up prior to initiating ART, particularly among children who were ineligible for ART. This represents substantially higher losses than those reported for children receiving ART [18]. The reasons for loss to follow-up are not completely understood, although a proportion due to unreported transfers and deaths are expected [25-27]. Resources should be directed towards retention to ensure that all children remain in care and are appropriately monitored.

Consistent with our findings at clinic enrollment, the profile of children initiating ART in urban and rural clinics improved with increasing program duration. This also has been observed in studies among adults in sub-Saharan Africa [5-7]. Outcomes were assessed after six months of treatment and mortality and loss to follow-up were within the ranges reported from other pediatric ART programs in sub-Saharan Africa [3,18]. Mortality appeared to be higher in the rural clinics and remained constant over time despite initiation at earlier stages of disease, perhaps due to increases in enrollment of younger children who are more likely to have high mortality [3,11]. In contrast, mortality in the urban clinic declined, a pattern which has been observed among adults initiating treatment in South Africa, and was attributed to improvements in the clinical profile of individuals initiating treatment [7]. However, in this study, greater improvements were observed in the rural clinics without any decrease in mortality. As predictors of mortality over time were not evaluated and the completeness of patient tracing in either location is unknown, we cannot determine whether the observed decrease in mortality in the urban clinic was in fact due to better survival resulting from improved clinical status at ART initiation or differential ascertainment of deaths in the clinics.

Loss to follow-up after ART initiation in the urban clinics remained stable over time. In the rural clinics losses increased, a pattern that has also been observed in adult programs [7,8]. This may be due to the large increase in enrollment over time, thereby reducing the ability of clinic staff to monitor and manage the patient load, or to the increasing distances patients travelled to the clinic, which was found to be associated with higher loss to follow-up in this population [28].

There were several limitations to this study. As the study was based on data abstracted from medical records, all relevant information was not available, and the data that were available were not complete, particularly for the anthropometric and laboratory measures. The completeness of the data was not associated with disease severity but was associated with age; younger children were more likely to have incomplete data, perhaps due to the increased difficulties in obtaining blood specimens from infants. The laboratory measures were performed more consistently in later years of program implementation, particularly in the rural clinics as the supply of reagents became more secure. In addition, these clinics, while typical of HIV clinics and subject to the same challenges and constraints as other clinics in this setting, may not be representative of all pediatric clinics in rural and urban areas of Zambia or other countries in this region.

## Conclusions

Progress has been made in the care of HIV-infected children in both urban and rural clinics. However, continued efforts are needed as many children continue to enter care at a late stage of disease and thus are not receiving the full benefits of treatment. As programs continue to expand, emphasis needs to shift to earlier identification of HIV-infected and exposed children, through promotion of counseling and testing, coordination between maternal and child health programs, and retention of children in care.

## Competing interests

The authors declare that they have no competing interests.

## Authors' contributions

CGS conceived the study, performed the data extraction and analysis and led the writing of the manuscript. CB-M supervised the study in Lusaka, Zambia, and participated in the writing of the manuscript. JHvD supervised the study in Macha, Zambia, and participated in the writing of the manuscript. MC supervised the study in Mukinge, Zambia, and participated in the writing of the manuscript. BT participated in the writing of the manuscript. WJM conceived the study, supervised the study in the US and participated in the writing of the manuscript. All authors have read and approved the final manuscript.

## Pre-publication history

The pre-publication history for this paper can be accessed here:

http://www.biomedcentral.com/1471-2431/10/54/prepub
